# Review article: Early steroid administration for traumatic haemorrhagic shock: A systematic review

**DOI:** 10.1111/1742-6723.14129

**Published:** 2022-11-08

**Authors:** Joseph P Hogarty, Morgan E Jones, Karishma Jassal, Daniel T Hogarty, Biswadev Mitra, Andrew A Udy, Mark C Fitzgerald

**Affiliations:** ^1^ Trauma Service The Alfred Hospital Melbourne Victoria Australia; ^2^ National Trauma Research Institute Melbourne Victoria Australia; ^3^ Department of Hyperbaric and Intensive Care Medicine The Alfred Hospital Melbourne Victoria Australia; ^4^ Emergency and Trauma Centre The Alfred Hospital Melbourne Victoria Australia; ^5^ School of Public Health and Preventive Medicine Monash University Melbourne Victoria Australia; ^6^ Australian and New Zealand Intensive Care Research Centre Monash University Melbourne Victoria Australia

**Keywords:** haemorrhagic shock, steroids, trauma

## Abstract

Haemorrhagic shock after trauma is a leading cause of death worldwide, particularly in young individuals. Despite advances in trauma systems and resuscitation strategies, mortality from haemorrhagic shock has not declined over the previous two decades. A proportion of shocked trauma patients may experience a deficiency of cortisol relative to the severity of their injury. The benefit of exogenous steroid administration in patients suffering haemorrhagic shock as a result of injury is unclear. A systematic review of four databases (Ovid Medline, Ovid Embase, Cochrane, Scopus) was undertaken. Inclusion and exclusion criteria were pre‐determined and two reviewers independently screened the articles with disagreements arbitrated by a third reviewer. The primary outcome variable was 28‐day mortality. Quality of studies were assessed using the Cochrane‐risk‐of‐bias (RoB 2) tool. Of the 2919 studies yielded by the search strategy, 1274 duplicates were removed and 1645 screened on title and abstract. After the full text of 33 studies were assessed, two articles were included. Both studies were over 30 years old with small numbers of participants and with primary outcomes not including mortality. Of the data available, no statistically significant difference in mortality was detected. Hospital length of stay, reversal of shock or adverse events were not reported. Both studies were at risk of bias. There are no high quality or recent studies in the English literature investigating the use of steroids for haemorrhagic shocked trauma patients. PROSPERO: CRD42021239656.

## Graphical abstract

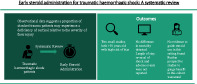

**Key findings**

- Observational data suggests a proportion of shocked trauma patients may experience a deficiency of cortisol relative to the severity of their injury.
- Our systematic review of the literature identified only two small studies, both over 30 years old at high risk of bias. No difference in mortality was detected, length of stay, reversal of shock and adverse events were not reported.
- Our review revealed no evidence to guide steroid use in the setting of traumatic hemorrhagic shock. Further prospective studies to gauge benefit from steroid use in this cohort is warranted.

## Introduction

Haemorrhagic shock is a life‐threatening condition of inadequate organ perfusion as a result of circulatory failure secondary to blood loss. Traumatic injury results in an estimated 60 000 deaths per year in the USA and 1.5 million deaths worldwide.^1^ This represents nearly 75 million years of lost life annually because of trauma's disproportionate effect on younger people.^1^, ^2^, ^3^ Trauma is the leading cause of mortality among people aged less than 44 years^4^ in the USA, and haemorrhagic shock contributes to 30–40% of these deaths.^5^

Research in the mid‐1970s highlighted the importance of timely haemostatic and resuscitative interventions.^6^ Trauma systems were developed to decrease the time to definitive care and improve outcomes for trauma patients. Modern damage control resuscitation strategies continue to focus on early haemostasis and correcting coagulopathy.^1^

Despite this, mortality rates among patients who present with shock have remained static since 2000.^7^, ^8^ While a large proportion of these deaths occur prehospital, a significant number of deaths are due to ongoing shock in the first 24 h of admission as well as associated subsequent deaths secondary to multiple organ dysfunction and infection.^5^

Profound hypovolemia and shock results in tissue hypoperfusion, neuroendocrine stress response and activation of the coagulation cascade producing an inflammatory pathway referred to as the systemic inflammatory response syndrome (SIRS). This causes cellular and organ damage and contributes to ongoing haemodynamic instability.^9^ Trauma represents the most common cause of non‐septic SIRS^10^ and consequently, the role of the hypothalamic–pituitary–adrenal axis is of interest.

Prospective observational studies have demonstrated that a significant proportion of haemorrhagic shocked trauma patients experience a deficiency of cortisol relative to the severity of their injury early in their resuscitation.^11^, ^12^ Reduced cortisol levels are seen to have a significant association with mortality.^11^, ^12^ Furthermore, levels are seen to increase throughout admission in survivors and decrease throughout admission for non‐survivors, with preserved levels of ACTH indicating an adrenal origin of reduced responsiveness.^12^

Numerous animal studies over the last century have established the importance of cortisol in the body's response to stress and haemorrhage.^13^, ^14^ Studies have been performed on a variety of animals utilising a wide range of methods for experimental trauma, with different steroids and doses administered to the subjects. Many demonstrate organ protection and mortality benefit when glucocorticoids are provided at the time of the experimental model of traumatic shock.^15^, ^16^, ^17^, ^18^, ^19^, ^20^, ^21^, ^22^, ^23^

Available experimental and observational data suggest a possible cortisol deficiency and potential utility of exogenous steroids in patients suffering traumatic haemorrhagic shock. Owing to the prevalence of traumatic haemorrhagic shock, benefit from a cheap and available drug would have a large impact. There appear to have been no published reviews to date examining the effectiveness of early administration of steroids to traumatic haemorrhagic shock patients. This systematic review was undertaken to determine the level of evidence supporting early steroid administration for traumatic haemorrhagic shock and its effect on mortality.

## Methods

This systematic review was performed as recommended in the Preferred Reporting Items for Systematic Review and Meta‐Analyses statement.^24^

### Literature search

Four electronic databases (Ovid Medline, Ovid Embase, Cochrane and Scopus) were searched systematically from inception to 2 March 2021. A preliminary search was conducted of Medline and Embase and analysis of text words and subject headings was performed to develop the final search strategy. In addition, reference lists of included articles and relevant reviews were manually searched for appropriate studies. A detailed description of the search strategy including all the search terms is included in Appendix S1.

### Inclusion and exclusion criteria

Only human studies and studies published in English were included. Papers with study populations (or included with sub‐analysis) of subjects with traumatic haemorrhagic shock to whom steroids were administered in the early phase were included. The early phase was defined as <24 h from time of injury or outlined as the ‘resuscitative phase’ by the paper's authors. Definition of shock was at the discretion of the authors of the individual trials. Administration of any type or dose of intravenous corticosteroids including dexamethasone, prednisolone or methylprednisolone was accepted. Paediatric studies, reports on patients with pre‐existing adrenal impairment, studies in which relevant outcomes were not reported and studies in which only measurements of cortisol levels were performed rather than the administration of steroids were excluded.

Randomised controlled trials, prospective and retrospective observational studies as well as case series were included. Reviews, including systematic reviews and meta‐analyses were excluded as well as opinion pieces.

### Study selection

Two reviewers (JPH and MEJ) independently screened the titles and abstracts of the studies to assess each article's suitability for inclusion. Any inconsistencies in screening were discussed and resolved in consultation with a third reviewer (KJ). Reference lists of relevant reviews and included full text studies were screened for suitable studies.

### Outcomes

The primary outcome measure was 28‐day mortality. Shorter term mortality was accepted if 28‐day mortality was not available. Secondary outcomes measured were early reversal of shock at Day 2 and reversal of shock at Day 7, time to shock reversal, length of stay in hospital and ICU, 90‐day mortality and ICU and in‐hospital mortality. Adverse outcomes of administration of steroids included gastrointestinal bleeding, hypernatraemia, sepsis/infections and hyperglycaemia. All studies meeting inclusion criteria were included in analysis. Study characteristics were entered into tables to compare against planned groups for each synthesis.

Subgroups included were (i) high dose *vs* low dose steroids; (ii) penetrating *vs* blunt trauma; (iii) duration of treatment: short ≤4 days *vs* long >4 days; and (iv) the type of steroid used.

### Statistical analysis

Data were collected independently by two reviewers (JPH and MEJ) and any inconsistencies were discussed and reviewed in conjunction with a third reviewer (KJ).

Dichotomous variables were planned to be analysed using the Mantel–Haenszel method and expressed as risk ratios (RRs). Continuous variables were to be analysed using the inverse variance random‐effects model and expressed as mean differences. A *P*‐value of <0.05 was used for statistical significance.

Heterogeneity was to be assessed using the Paule and Mandel estimation method as well as visual inspection of forest plots.

### Risk of bias assessments

RoB 2: Revised Cochrane Risk of Bias Assessment Tool^25^ was performed on included studies independently by two authors (JPH and MEJ) with no affiliations to the trials. Disagreements were discussed and resolved by consensus and in conjunction with a third reviewer (KJ). Effect of assignment to intended intervention was assessed including domains reviewing the randomisation process, adherence to intended interventions, completeness of outcome data, measurement and selection. Overall risk of bias was only judged as low if all domains returned a low risk of bias result.

### Grading the quality of evidence

Grading of recommendations, assessment, development and evaluations (GRADE) was used to assess the quality of evidence for each outcome. Risk of bias in individual studies, publication bias, precision, consistency and directness were assessed to give an overall confidence rating of effect estimates for each outcome.

## Results

**Figure 1:**
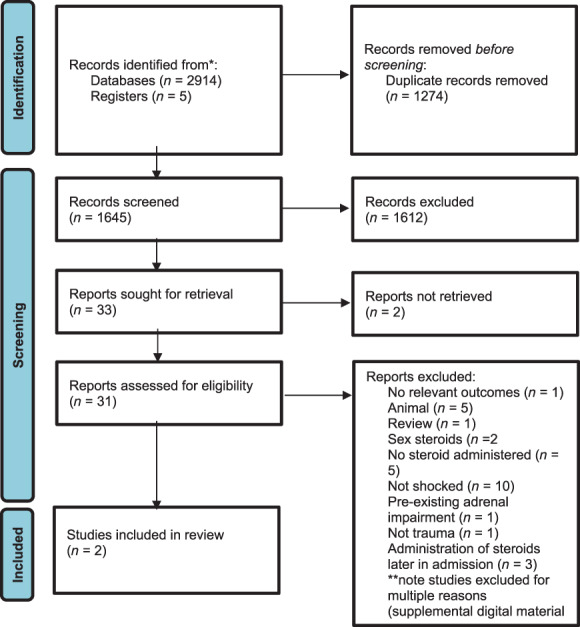
PRISMA 2020 flow diagram illustrating the number of studies identified by the search and the stages in which they were chosen and eliminated.

The search strategy yielded 2914 records. An additional five records were identified through reference lists of relevant reviews and included studies. One thousand two hundred and seventy‐four duplicates were removed. One thousand six hundred and forty‐five studies were screened on title and abstract by the two reviewers. Thirty‐three papers were identified for assessment of full text for eligibility resulting in inclusion of two randomised controlled trials (Fig. 1; Table 1). Reasons for exclusion of 31 papers on full text are included in Figure 1 and Appendix S2.

**TABLE 1:** Grade assessment of included studies

Grade domains	Judgements	Concerns about certainty domains
Methodological limitations of studies	Lucas and Ledgerwood's trial was at an overall ‘high risk of bias’ with concerns regarding the randomisation process and selection of results. Schumer and Nyhus' trial was at ‘some risk of bias’ with concerns regarding the randomisation process and selection of results.	Serious
Indirectness	Mortality was reported as a secondary outcome, timeframe of measurement was not reported. Patient groups were resuscitated with large volume crystalloid standard at the time of the study's publication but no longer in practice. Similarly massive dose steroids were utilised at doses no longer used for treatment of shock.	Serious
Imprecision	The combined number of patients in both trials was 164. Both revealed non‐significant results.	Serious
Inconsistency	Both trials revealed trends in different directions. Lucas and Ledgerwood's trial revealed a non‐significant trend towards increased mortality in the steroid treated group whereas Schumer and Nyhus' trial revealed a trend towards increased survival in the steroid treated group.	Serious
Publication bias	Both negative and positive trials were published and as such suspicion of publication bias is low.	Not serious

The first by Lucas and Ledgerwood in 1981 randomised 114 shocked surgical trauma patients from December 1977 to April 1980 treated within the Department of Surgery, Wayne State University, Detroit to receive either supplemental massive dose methylprednisolone or no steroids based on the last digit of their patient identifying number.^26^ Fifty‐four patients were administered supplemental steroids and sixty were not. The criteria for inclusion were ‘…multiple transfusions of blood, survived operation and …successfully stabilised in the immediate postoperative period’. There was no data related to other epidemiologic criteria (i.e. age, Injury Severity Score, Glasgow Coma Score, nature of injuries). However, the authors state that ‘…the two groups were comparable for age, type of injury, location of injury and number of organs injured’.

Methylprednisolone 1 g was provided in Phase 1, defined by the researchers as the period of active haemorrhage (admission until end of operation for haemorrhage control). In this period, an average of 13.1 units of blood, 11.7 L of balanced salt solution and 800 ml of FFP were transfused in similar quantities between the two groups. A further 15 mg/kg of methylprednisolone was provided to the intervention group over the next 3 days.

The primary outcome for the study were measures of pulmonary function. Differences in mortality was a secondary outcome. There was no significant mortality difference (7/54 in steroid supplemented patients compared to 2/60 control patients, *P* < 0.10, *χ* ^2^ = 3.6 as reported in trial). Ventilator support was required for 5.1 days in the steroid‐supplemented group *vs* 3.0 in non‐supplemented group (*P* < 0.10)^26^ (Table 2). The study reported large crystalloid volumes administered, which was representative of resuscitation at that time and which may have confounded outcomes including survival from pulmonary and neurological injury.^27^

**TABLE 2:** Characteristics of included studies

	Lucas and Ledgerwood	Schumer and Nyhus
Year	1981	1970
Study design	Randomised controlled trial	Double blind randomised controlled trial
Randomisation method	Last digit of patient identifier, not blinded, no placebo	Not reported
Number of patients	114	50
Age	Not reported	Not reported
Injury Severity Score	Not reported	Not reported
GCS	Not reported	Not reported
Traumatic brain injury	Not reported	Not reported
Definition of ‘shock’	SBP <80 mmHg	Based on clinical signs of peripheral vascular collapse (cold clammy skin, pallor, sweating, HR >120, SBP <80 and oliguria)
Steroid	Methylprednisolone 1 g followed by 15 mg/kg over next 3 days	Dexamethasone 1 mg/kg
Time of infusion	Phase 1 (active haemorrhage, admission to end of operation for bleeding control)	Not reported, steroids given to patients who did not respond to volume replacement
Mortality	7/52 steroid *vs* 2/60 control (not statistically significant *P* < 0.10, *χ* ^2^ = 3.6) (timeframe not reported)	Mortality rate 20% steroid *vs* 40% control (statistical significance not reported – *χ* ^2^ 2.38, *P* = 0.12 when calculated from data) (raw numbers not reported, extrapolating from percentages) (timeframe not reported)
Reversal of shock	Not reported	Biochemical index (mean of lactate, phosphate and serum amino acids) returned to baseline 75 min earlier than control)
Hospital length of stay	Not reported	Not reported
ICU length of stay	Not reported	Not reported
Incidence of adverse events	Not reported	Not reported
Cochrane risk of bias assessment	High	Some concerns

GCS, Glasgow Coma Scale; HR, heart rate; SBP, systolic blood pressure.

Risk of bias assessment (RoB 2) looking at mortality revealed an overall ‘high risk of bias’. This arose from Domain 1 (randomisation process) relating to the allocation of patients via their patient identifier number and the lack of concealment associated with this. ‘Some risk of bias’ was also identified in Domain 5 (selection bias of reported result) in relation to the lack of clarity regarding when mortality was measured. Domains 2, 3 and 4 returned a low risk of bias result (Appendix S3).

In the second paper published in 1970, Schumer and Nyhus from the Department of Surgery, University of Illinois College of Medicine, randomised 50 haemorrhagic shocked trauma patients not responding to volume replacement with blood, Ringer's solution and sodium bicarbonate to receive supplemental dexamethasone or saline solution. It is not explicitly stated how many patients were in the experimental *vs* control group. 1 mg/kg of dexamethasone was provided to patients in the experimental group, the specific time or method of infusion was not reported.

A survival rate of 80% in the steroid treated group *vs* 60% in the control group was reported, the time point of determination of mortality was not mentioned in the text. Statistical significance was not reported. However, using the data provided the *χ* ^2^ statistic is 2.38 (*P* = 0.12). A ‘biochemical index’ consisting of the mean values of lactate, phosphate and serum amino acids returned to baseline 75 min earlier in steroid treated patients compared to the controls. Statistical significance related to this was not reported^28^ (Table 2). The study reported whole blood:Ringer's solution resuscitation in a ratio of 1:2, which in the same way may have affected outcomes including survival from pulmonary and neurological injury.

RoB 2 reviewing mortality demonstrated an overall ‘some risk of bias’ result. This arose from Domain 1 (randomisation process) because of the lack of information pertaining to the randomisation process and baseline characteristics of the patients. ‘Some risk of bias’ was also found in relation to Domain 5 (selection bias of reported result) in relation to the lack of clarity regarding when mortality was measured. Domains 2, 3, 4 returned ‘low risk of bias’ results (Appendix S4).

As a result of the limited number of studies identified, and a lack of clinically or statistical homogeneity, a pooled analysis was not undertaken.

GRADE assessment performed on the studies indicated a very low certainty in the evidence found (Table 2).

## Discussion

### Main findings

The effectiveness of early steroids during the management of traumatic haemorrhagic shock after trauma was assessed by two trials, both of which were old and missing key variables regarding the age of subjects, severity of trauma and nature of injuries. There was no reported statistically significant association with mortality, although both studies involved small numbers and mortality was not a primary outcome.^26^, ^28^ Adverse events were not reported in either study. Resuscitation strategies with large volume crystalloids, no longer recommended but consistent with practice at the time of publication, were employed. Both studies were at risk of bias determined by application of the Cochrane RoB 2 tool. As such, the evidence identified from this review was considered very‐low quality.

### Relationship with previous work

Consistent with trends in shock management at the time of publications,^29^ high‐dose steroids were utilised in the included trials. This trend fell out of favour in the 1980s with studies in septic cohorts revealing no mortality benefit and an increased incidence of adverse outcomes.^30^ Recent large multi‐centre trials in septic cohorts utilising low dose steroids have demonstrated shortened time to resolution of shock, decreased time on mechanical ventilation and reduced time spent in ICU with no overall difference in mortality.^31^

Meta‐analyses performed by the Society of Critical Care Medicine and European Society of Intensive Care Medicine examined 12 000 trauma patients provided with supplemental steroids of any dose. No statistically significant benefit or harm was found among the 12 000 patients included. No difference was found when stratified into low dose or high dose corticosteroid. No increased risk of superinfection or gastrointestinal bleeding was identified.^32^, ^33^ This patient cohort was not specific to haemorrhagic shock and in fact a large number of these patients had suffered head injuries. The large multicentre CRASH1 trial in 2004 found an increased mortality when providing high dose steroids to head injured patients.^34^ The pathophysiological rationale for providing steroids to this cohort is very different – attempting to counteract post‐traumatic neuronal degeneration.

The HYPOLYTE study by Roquilly *et al*. provided low dose hydrocortisone or placebo within 36 h to ICU trauma patients, not necessarily haemorrhagic shock, expected to be mechanically ventilated for >48 h.^35^ Steroids continued daily for 7 days, with doses ceased in those found not to be adrenally insufficient. 60% of patients were found to be hypotensive but this sub‐group was not analysed for outcomes. Overall, there were two deaths among the 150 randomised patients, both in the treatment arm. The study lacked sufficient power to reveal any difference in mortality rates. It did, however, demonstrate a reduction in the incidence of pneumonia as well as days of ventilation, days in ICU, time to noradrenaline withdrawal and development of ARDS in the treatment arm. No increase in gastrointestinal bleeding was noted in treated patients.^35^

Similarly, Chaari *et al*. compared a prospective cohort of ICU trauma patients provided with steroids with a retrospective control cohort (175 total). 45% of patients were hypotensive but this subgroup was not analysed separately for outcomes.^36^ There was a lower incidence of pulmonary embolism but no difference in ICU stay, duration of ventilation or mortality among treated patients.^36^

### Trials studying cortisol levels

The rationale behind providing supplemental steroids to shocked patients comes from data suggesting that critical illness induces a state of insufficient cortisol response relative to the insult. In the traumatic haemorrhagic shock setting this has been examined in a small number of prospective and retrospective papers. Stein *et al*. conducted a prospective observational study measuring the admission cortisol of 59 consecutive patients presenting to their trauma centre in the USA with haemorrhagic shock.^11^ They found that 17% (*n* = 10) of patients had what they defined as severe hyper‐acute adrenal insufficiency (SHAI) (<10 μg/dl) and 86% (*n* = 51) met their definition for relative hyper‐acute adrenal insufficiency (RHAI) (<25 μg/dl). 46 (78%) of patients had a time of injury available and blood was drawn an average of 56.6 (±26.3) min from this injury.^11^ Their analyses revealed an OR of 1.23 (95% CI 1.09–1.38; *P* = 0.001) indicating each 1 μg/dl decrease in cortisol level in this cohort was associated with a 23% increase in mortality within 24 h.

In a similar fashion, Joseph *et al*. tracked the levels of cortisol and other pituitary hormones of 42 haemorrhagic shock trauma patients at admission and over the first few days of their hospitalisation.^12^ Using the same parameters as Stein *et al*. their population had an incidence of 19% SHAI and 76.2% RHAI. Non‐survivors were found to have lower admission cortisol levels when compared with survivors. Interestingly, survivor cortisol levels increased throughout their admission (19.1 ± 6.7 pg/ml *vs* 8.1 ± 5.1 pg/ml, *P* = 0.003) whereas non‐survivors cortisol decreased significantly over time, perhaps suggesting a decompensated response (14.3 ± 7.1 μg/dl *vs* 6.3 ± 0.6 μg/dl; *P* = 0.03).^12^ Non‐survivor adrenocorticotropic hormone (ACTH) levels remained elevated despite dwindling cortisol suggesting an adrenal origin of reduced responsiveness.^12^, ^37^

Likewise, Barton *et al*. examined cortisol and ACTH levels but across the spectrum of trauma presentations.^37^ They found that cortisol levels rose with severity of injury from low‐to‐moderate severity, yet fell in the severely injured group. In this group as injury severity increased, cortisol levels fell in relation to ACTH – again suggesting the origin was adrenal insufficiency.^37^

Similarly, Cohn *et al*. assessed a variety of vasoactive mediators, including cortisol among trauma patients presenting to their facility with a systolic blood pressure <90.^38^ Patients adjudged to be in shock (sustained systolic blood pressure <90 mmHg and/or base deficit <−6 mEq/L) had a non‐significant, mean cortisol level of 6.69 μg/dl, lower than their non‐shocked counterparts.^38^ No temporal pattern was noted in serial cortisol levels over 2 h in this cohort. In a small retrospective review, Rushing *et al*. observed the cortisol level of 15 subjects presenting with haemorrhagic shock whom had their cortisol measured.^39^ 1 of 15 met the criteria level for SHAI as described above and 14/15 for RHAI in their cohort.^39^

Although there appears to be a reasonable prevalence of relative adrenal insufficiency (RAI) in early haemorrhagic shock trauma patients, the pathophysiology of cortisol differs in the acute inflammatory state. Normally free cortisol acts as the active glucocorticoid in the body but only represents 5–10% of total body cortisol; with the remainder bound mostly to corticotropin binding globulin (CBG) and to a lesser extent albumin.^40^ After an acute insult, levels of CBG are seen to decrease as does the activity of cortisol metabolising enzymes, both contributing to increased levels of biologically active free cortisol.^41^, ^42^ Working in a contradictory manner, peripheral tissue displays an increased resistance to cortisol in the acute phase adding further difficulty to assessment of an appropriate cortisol level.^42^, ^43^

As a result of these complicating factors, there is no consensus on diagnostic levels for a RAI. Studies use a variety of thresholds including a baseline cortisol <10/15/25 μg/dl or a decreased response to a synthetic ACTH stimulation test. This makes the interpretation of comparative results difficult.^10^

### Study implications

Injury resulting in haemorrhagic shock is a leading cause of mortality globally as well as economic burden through medical costs and loss of productivity.^7^, ^44^

This review revealed no recent or high‐quality studies to guide the use of early steroids in traumatic haemorrhagic shock patients. It is known that haemorrhagic shocked trauma patients experience ongoing haemodynamic instability related to SIRS. Although estimation of active cortisol in the acute state is difficult, there is evidence to suggest that a proportion of trauma patients, perhaps related to uncharacterised phenotypic variations, experience a deficiency in endogenous glucocorticoid in response to the severity of injury and the presence of haemorrhagic shock.^45^ As such, our review implies the need for high‐quality research in this area.

### Strengths and weaknesses

This review was conducted in accordance with the PRISMA statement. A research librarian was consulted to assist with developing the search strategy (Appendix S5) and a broad range of databases were searched as well as screening of reference lists of included articles and relevant reviews. Articles were screened independently by two reviewers. Included articles were independently assessed for risk of bias using the Cochrane risk of bias tool (RoB 2).

A small number of studies met the inclusion criteria highlighting the gap in the literature regarding steroid use in traumatic haemorrhagic shock patients. Identified studies were both over 30 years old and utilised high dose steroids and resuscitation strategies current at the time of publication but no longer in practice, making them of little relevance to present day management. As a result of the limited number of included studies, a pooled analysis was not undertaken.

## Conclusion

This review of over 2900 studies yielded only two studies, both over 30 years old with administration of very high steroid doses, small numbers of participants and incomplete results. Both were at risk of bias and of very low quality. As such, among haemorrhagic shocked adult trauma subjects there has been no high quality or recent studies examining the effect on mortality of early steroid supplementation. Given that steroids are cheap, ubiquitous and simple to administer and the magnitude of patients affected by traumatic haemorrhagic shock each year, further prospective studies to gauge benefit from steroid use in this cohort is warranted.

## Supporting information


**Appendix S1.** Database search strategy – MEDLINE.

**Appendix S2**. List of citations identified on title and abstract and reason for exclusion or inclusion.

**Appendix S3.** RoB 2 Lucas and Ledgerwood – Mortality.

**Appendix S4**. RoB 2 Schumer and Nyhus ‐ Mortality.

**Appendix S5**. PRISMA checklist.

## Data Availability

The data that support the findings of this study are available from the corresponding author upon reasonable request.
