# Supplementary material for: Review article: Early steroid administration for traumatic haemorrhagic shock: A systematic review
Source: Emerg Med Australas. 2022 Nov 8;35(1):6–13. doi: 10.1111/1742-6723.14129 (PMC10100146; doi:10.1111/1742-6723.14129)
Supplement: Supplementary file 1 — Appendix S1. Database search strategy – MEDLINE. [file EMM-35-6-s002.docx]

**Appendix S1. Database search strategy - MEDLINE**

| **#** | **Searches** |
| --- | --- |
| 1 | Steroids/ Corticosteroid/ glucocorticoids/ Dexamethasone/ Hydrocortisone/ Methylprednisolone/ Methylprednisolone hemisuccinate/ Prednisolone/ Prednisone/ Cortisone |
| 2 | (Steroid* or corticosteroid* or glucocorticoid* or dexamethasone or hydrocortisone or methylprednisolone* or prednis* or cortisone).mp. |
| 3 | (Predonin* or decortin or nisolone or cortef or chronocort or medrol or fortecortin or decadron or dexasone or dexascheroson or oradexon).mp. |
| 4 | Hypovolemic shock/ Haemorrhagic shock/ Traumatic shock/ |
| 5 | (Traumatic adj2 shock or h?em*or*ag* shock or hypovol?emic shock).mp. |
| 6 | (1 or 2 or 3) and (4 or 5) |
| 7 | exp animals/ not humans.sh. |
| 8 | 6 not 7 |
| 9 | Limit 8 to English language |

- Search performed by Dr Joseph Hogarty, with consultation from Lorena Romero Alfred Health librarian
- Search performed from inception until 2^nd^ March 2021
- Reference list of reviews and relevant papers hand searched for relevant studies
- Search results uploaded into ‘Eppi Reviewer 4’ and screened
- Same search strategy with minor search system alterations used for EMBASE, COCHRANE and SCOPUS
