# Supplementary material for: Review article: Early steroid administration for traumatic haemorrhagic shock: A systematic review
Source: Emerg Med Australas. 2022 Nov 8;35(1):6–13. doi: 10.1111/1742-6723.14129 (PMC10100146; doi:10.1111/1742-6723.14129)
Supplement: Supplementary file 2 — Appendix S2. List of citations identified on title and abstract and reason for exclusion or inclusion. [file EMM-35-6-s003.docx]

**Appendix S2. List of citations identified on title and abstract and reason for exclusion or inclusion**

| Effects of glucocorticoids on ventricular performance and capillary permeability during hemorrhagic shock | - Animal study |
| --- | --- |
| Pulmonary function in patients with multiple trauma and associated severe head injury. | - Limited number were shocked - No relevant outcomes |
| Catecholamines in shock | - Review paper |
| Peripheral vascular actions of glucocorticoids and their relationship to protection in circulatory shock. | - Animal |
| Glucocorticoid-Induced Protection in Circulatory Shock: Role of Reticuloendothelial System Function | - Animal |
| Dehydroepiandrosterone: An inexpensive steroid hormone that decreases the mortality due to sepsis following trauma-induced hemorrhage | - Sex steroid not glucocorticoids |
| Sex steroids regulate pro- and anti-inflammatory cytokine release by macrophages after trauma-hemorrhage | - Sex steroid not glucorticocoids |
| Ulinastatin-a newer potential therapeutic option for multiple organ dysfunction syndrome | - Not steroid |
| Treatment of experimental hemorrhagic shock: Comparison of the effects of ACTH-(1-24), methylprednisolone, aprotinin and norepinephrine | - Animal |
| The use of a low dose hydrocortisone to prevent pulmonary embolism in patients with multiple trauma. | - Trauma ICU patients (doesn’t say when steroids given, but says 100mg 8 hourly for seven days) - Eighty (45% hypotensive), hypotension/shock not sub-analysed for outcomes |
| Does low-dose hydrocortisone therapy prevent ventilator-associated pneumonia in trauma patients? | - Same cohort as above study |
| Corticosteroids for fat embolism after multiple fractures | - Case report where steroids not given and review |
| The use of intravenous hydrocortisone in hemorrhagic shock | - Animal |
| Septic complications of corticosteroid administration after central nervous system trauma | - Exclusively head injury patients and infective complications - Not shocked |
| HYPOLYTE study | - Hydrocortisone within 36 hours, ICU trauma patients - Doesn’t sub-analyse shocked patients, |
| Adrenocortical suppression in multiply injured patients: A complication of etomidate treatment | - 9 patients, 6 etomidate, not shocked |
| Mapping the Steroid Response to Major Trauma From Injury to Recovery: A Prospective Cohort Study. | - Steroid not given |
| Acute pulmonary insufficiency. Treatment in Vietnam casualties. | - Not hypovolaemic shock |
| Empiric stress dose steroids in trauma patients: A case report of hypopituitarism in traumatic hemorrhage | - Established hypopitiutarism |
| Elevation of blood pressure by intravenous use of hydrocortisone in hemorrhagic shock. | - Not trauma |
| Hydrocortisone increases the sensitivity to 1-adrenoceptor stimulation in humans following hemorrhagic shock (Hoen et. Al.) | - ICU trauma patients - Not all shock (not sub-analysed), not early |
| Fat embolism in severely injured patients | - Not shocked |
| The use of corticosteroids in the treatment of shock. Lillehei et. Al. | - Abstract or full text not available |
| Corticosteroid after etomidate in critically ill Patients: A randomized controlled Trial | - Not trauma, not shock |
| **Corticosteroid effect on biochemical parameters of human oligemic shock** | - Included – met pre-determined criteria |
| Relative adrenal insufficiency: The impact of hemorrhagic shock | - Steroids not given |
| Incidence and outcomes of critical illness-related corticosteroid insufficiency in trauma patients. | - Retrospective review of who received steroids, not shock and not early steroids |
| Critical illness-related corticosteroid insufficiency after multiple traumas: a multicenter, prospective cohort study. | - Steroid not given |
| Acute Adrenal Insufficiency May Affect Outcome in the Trauma Patient | - Not early, tested for adrenal insufficiency |
| **Pulmonary response of massive steroids in seriously injured patients** | - Included – met pre-determined criteria |
| High-dose corticosteroids in thoracic trauma | - Not shocked patients |
| Relative adrenal insufficiency: an identifiable entity in nonseptic critically ill patients? | - Retrospective, look at patients who had received corticotropin stimulation test in their ICU - Non-septic patients – not necessarily trauma or haemorrhagic shock - Some were treated with steroids at discretion of clinician, not early steroids |
| Clinical Characteristics of Trauma Patients  Requiring Hydrocortisone Treatment for  Refractory Hypotension | - Not early steroids and not specifying if shocked or reason for shock – discussion states that most were probably patients who developed sepsis |
