# Supplementary material for: Review article: Early steroid administration for traumatic haemorrhagic shock: A systematic review
Source: Emerg Med Australas. 2022 Nov 8;35(1):6–13. doi: 10.1111/1742-6723.14129 (PMC10100146; doi:10.1111/1742-6723.14129)
Supplement: Supplementary file 3 — Appendix S3. RoB 2 Lucas and Ledgerwood – Mortality. [file EMM-35-6-s005.docx]

Revised Cochrane risk-of-bias tool for randomized trials (RoB 2)

TEMPLATE FOR COMPLETION

Edited by Julian PT Higgins, Jelena Savović, Matthew J Page, Jonathan AC Sterne
on behalf of the RoB2 Development Group

**Version of 22 August 2019**

The development of the RoB 2 tool was supported by the MRC Network of Hubs for Trials Methodology Research (MR/L004933/2- N61), with the support of the host MRC ConDuCT-II Hub (Collaboration and innovation for Difficult and Complex randomised controlled Trials In Invasive procedures - MR/K025643/1), by MRC research grant MR/M025209/1, and by a grant from The Cochrane Collaboration.


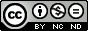


This work is licensed under a [Creative Commons Attribution-NonCommercial-NoDerivatives 4.0 International License](http://creativecommons.org/licenses/by-nc-nd/4.0/).

| **Study details**   \| **Reference** \| [Lucas CE, Ledgerwood AM. Pulmonary response of massive steroids in seriously injured patients. *Ann Surg*. 1981;194:256–261.](http://paperpile.com/b/13ZJht/ySolD) \| \| --- \| --- \|   **Study design**   \| X \| Individually-randomized parallel-group trial \| \| --- \| --- \| \| ⬜ \| Cluster-randomized parallel-group trial \| \| ⬜ \| Individually randomized cross-over (or other matched) trial \|   **For the purposes of this assessment, the interventions being compared are defined as**   \| Experimental: \| Methylprednisolone \| Comparator: \| Nil methylprednisolone \| \| --- \| --- \| --- \| --- \|  \| **Specify which outcome is being assessed for risk of bias** \| Mortality \| \| --- \| --- \|  \| **Specify the numerical result being assessed.** In case of multiple alternative analyses being presented, specify the numeric result (e.g. RR = 1.52 (95% CI 0.83 to 2.77) and/or a reference (e.g. to a table, figure or paragraph) that uniquely defines the result being assessed. \| 7/54 deaths in experimental group vs 2/60 in comparator group RR 3.89 (chi square 3.6; p = <0.10) \| \| --- \| --- \|   **Is the review team’s aim for this result…?**   \| X \| to assess the effect of *assignment to intervention* (the ‘intention-to-treat’ effect) \| \| --- \| --- \| \| ⬜ \| to assess the effect of *adhering to intervention* (the ‘per-protocol’ effect) \|   **If the aim is to assess the effect of *adhering to intervention***, select the deviations from intended intervention that should be addressed (at least one must be checked):  ⬜ occurrence of non-protocol interventions  ⬜ failures in implementing the intervention that could have affected the outcome  ⬜ non-adherence to their assigned intervention by trial participants  **Which of the following sources were obtained to help inform the risk-of-bias assessment? (tick as many as apply)**  X Journal article(s) with results of the trial  ⬜ Trial protocol  ⬜ Statistical analysis plan (SAP)  ⬜ Non-commercial trial registry record (e.g. ClinicalTrials.gov record)  ⬜ Company-owned trial registry record (e.g. GSK Clinical Study Register record)  ⬜ “Grey literature” (e.g. unpublished thesis)  ⬜ Conference abstract(s) about the trial  ⬜ Regulatory document (e.g. Clinical Study Report, Drug Approval Package)  ⬜ Research ethics application  ⬜ Grant database summary (e.g. NIH RePORTER or Research Councils UK Gateway to Research)  ⬜ Personal communication with trialist  ⬜ Personal communication with the sponsor |
| --- | --- | --- | --- | --- | --- | --- | --- | --- | --- | --- | --- | --- | --- | --- | --- | --- | --- | --- | --- | --- |

## Risk of bias assessment

Responses underlined in green are potential markers for low risk of bias, and responses in red are potential markers for a risk of bias. Where questions relate only to sign posts to other questions, no formatting is used.

**Domain 1: Risk of bias arising from the randomization process**

| **Signalling questions** | **Comments** | **Response options** |
| --- | --- | --- |
| **1.1 Was the allocation sequence random?** | No. Patients were allocated based on the last digit of their patient identifier number.  Probably no | N |
| **1.2 Was the allocation sequence concealed until participants were enrolled and assigned to interventions?** |  | PN |
| **1.3 Did baseline differences between intervention groups suggest a problem with the randomization process?** | Probably no. Table of a study compares the admission blood pressure of the two groups: 79 +/- 4 in experimental group and 76 +/- 3 in comparator group and states no significant difference. It also states no significant difference in age, type of injury, location of injury and number of organs injured. Other baseline characteristics such as presence of head injury/GCS was not included. | PN |
| **Risk-of-bias judgement** | High | High |
| Optional: What is the predicted direction of bias arising from the randomization process? | Unpredictable | NA / Favours experimental / Favours comparator / Towards null /Away from null / Unpredictable |

Domain 2: Risk of bias due to deviations from the intended interventions (*effect of assignment to intervention*)

| **Signalling questions** | **Comments** | **Response options** |
| --- | --- | --- |
| **2.1. Were participants aware of their assigned intervention during the trial?** | No. However due to being shocked at admission would not have been able to process this information at the time however they could have been aware of receiving the intervention at the time of subsequent doses over the coming days (three days).  Yes. No mention of blinding or placebo indicates that the carers and people delivering the trial would have known which patients were receiving the intervention | PN |
| **2.2. Were carers and people delivering the interventions aware of participants' assigned intervention during the trial?** |  | Y |
| **2.3. If Y/PY/NI to 2.1 or 2.2: Were there deviations from the intended intervention that arose because of the trial context?** | Probably no. Average intervention doses were mentioned but did not specifically mention if these were complete doses. There was no mention of the intervention being administered to the comparator group. | PN |
| **2.4 If Y/PY to 2.3: Were these deviations likely to have affected the outcome?** | N/A | NA |
| **2.5. If Y/PY/NI to 2.4: Were these deviations from intended intervention balanced between groups?** | N/A | NA |
| **2.6 Was an appropriate analysis used to estimate the effect of assignment to intervention?** | Yes. Not explicitly stated but implied that all experimental group received full course of therapy (3 day course). All patients whose records were available were included in the analysis | PY |
| **2.7 If N/PN/NI to 2.6: Was there potential for a substantial impact (on the result) of the failure to analyse participants in the group to which they were randomized?** | N/A | NA |
| **Risk-of-bias judgement** | Low | Low |
| Optional: What is the predicted direction of bias due to deviations from intended interventions? | NA | NA |

Domain 2: Risk of bias due to deviations from the intended interventions (*effect of adhering to intervention*)

| **Signalling questions** | **Comments** | **Response options** |
| --- | --- | --- |
| **2.1. Were participants aware of their assigned intervention during the trial?** |  | Y / PY / PN / N / NI |
| **2.2. Were carers and people delivering the interventions aware of participants' assigned intervention during the trial?** |  | Y / PY / PN / N / NI |
| **2.3. [If applicable:] If Y/PY/NI to 2.1 or 2.2: Were important non-protocol interventions balanced across intervention groups?** |  | NA / Y / PY / PN / N / NI |
| **2.4. [If applicable:] Were there failures in implementing the intervention that could have affected the outcome?** |  | NA / Y / PY / PN / N / NI |
| **2.5. [If applicable:] Was there non-adherence to the assigned intervention regimen that could have affected participants’ outcomes?** |  | NA / Y / PY / PN / N / NI |
| **2.6. If N/PN/NI to 2.3, or Y/PY/NI to 2.4 or 2.5: Was an appropriate analysis used to estimate the effect of adhering to the intervention?** |  | NA / Y / PY / PN / N / NI |
| **Risk-of-bias judgement** |  | Low / High / Some concerns |
| Optional: What is the predicted direction of bias due to deviations from intended interventions? |  | NA / Favours experimental / Favours comparator / Towards null /Away from null / Unpredictable |

Domain 3: Missing outcome data

| **Signalling questions** | **Comments** | **Response options** |
| --- | --- | --- |
| **3.1 Were data for this outcome available for all, or nearly all, participants randomized?** | No. 130 patients were randomised treated between December 1977 through April 1980. Due to closure of the hospital and problems with transfer of records to the new hospital patients treated after January 30 1980 did not have records available and were not included. This consisted of 16 patients. | N |
| **3.2 If N/PN/NI to 3.1: Is there evidence that the result was not biased by missing outcome data?** | Yes. Any patient treated after January 30 1980 was not included in essence shortening the trial period rather than introducing bias. | Y |
| **3.3 If N/PN to 3.2: Could missingness in the outcome depend on its true value?** |  |  |
| **3.4 If Y/PY/NI to 3.3: Is it likely that missingness in the outcome depended on its true value?** |  |  |
| **Risk-of-bias judgement** | Low | Low |
| Optional: What is the predicted direction of bias due to missing outcome data? | NA | NA |

Domain 4: Risk of bias in measurement of the outcome

| **Signalling questions** | **Comments** | **Response options** |
| --- | --- | --- |
| **4.1 Was the method of measuring the outcome inappropriate?** | Yes. Mortality | N |
| **4.2 Could measurement or ascertainment of the outcome have differed between intervention groups?** | No. | N |
| **4.3 If N/PN/NI to 4.1 and 4.2: Were outcome assessors aware of the intervention received by study participants?** | Yes | Y |
| **4.4 If Y/PY/NI to 4.3: Could assessment of the outcome have been influenced by knowledge of intervention received?** | No | N |
| **4.5 If Y/PY/NI to 4.4: Is it likely that assessment of the outcome was influenced by knowledge of intervention received?** |  | N |
| **Risk-of-bias judgement** | Low | Low |
| Optional: What is the predicted direction of bias in measurement of the outcome? | N/A | NA |

Domain 5: Risk of bias in selection of the reported result

| **Signalling questions** | **Comments** | **Response options** |
| --- | --- | --- |
| **5.1 Were the data that produced this result analysed in accordance with a pre-specified analysis plan that was finalized before unblinded outcome data were available for analysis?** | Not enough information | NI |
| **Is the numerical result being assessed likely to have been selected, on the basis of the results, from...** |  |  |
| **5.2. ... multiple eligible outcome measurements (e.g. scales, definitions, time points) within the outcome domain?** | NI. No time point on mortality measurement | NI |
| **5.3 ... multiple eligible analyses of the data?** | NI | NI |
| **Risk-of-bias judgement** | Some concerns | Some concerns |
| Optional: What is the predicted direction of bias due to selection of the reported result? |  | Unpredictable |

Overall risk of bias

| **Risk-of-bias judgement** | High | High |
| --- | --- | --- |
| Optional: What is the overall predicted direction of bias for this outcome? | Unpredictable | Unpredictable |


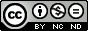


This work is licensed under a [Creative Commons Attribution-NonCommercial-NoDerivatives 4.0 International License](http://creativecommons.org/licenses/by-nc-nd/4.0/).
