# Supplementary material for: Review article: Early steroid administration for traumatic haemorrhagic shock: A systematic review
Source: Emerg Med Australas. 2022 Nov 8;35(1):6–13. doi: 10.1111/1742-6723.14129 (PMC10100146; doi:10.1111/1742-6723.14129)
Supplement: Supplementary file 4 — Appendix S4. RoB 2 Schumer and Nyhus ‐ Mortality. [file EMM-35-6-s001.docx]

Revised Cochrane risk-of-bias tool for randomized trials (RoB 2)

TEMPLATE FOR COMPLETION

Edited by Julian PT Higgins, Jelena Savović, Matthew J Page, Jonathan AC Sterne
on behalf of the RoB2 Development Group

**Version of 22 August 2019**

The development of the RoB 2 tool was supported by the MRC Network of Hubs for Trials Methodology Research (MR/L004933/2- N61), with the support of the host MRC ConDuCT-II Hub (Collaboration and innovation for Difficult and Complex randomised controlled Trials In Invasive procedures - MR/K025643/1), by MRC research grant MR/M025209/1, and by a grant from The Cochrane Collaboration.


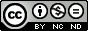


This work is licensed under a [Creative Commons Attribution-NonCommercial-NoDerivatives 4.0 International License](http://creativecommons.org/licenses/by-nc-nd/4.0/).

| **Study details**   \| **Reference** \| [Schumer W, Nyhus LM. Corticosteroid effect on biochemical parameters of human oligemic shock. *Arch Surg*. 1970;100:405–408.](http://paperpile.com/b/13ZJht/jt6Ga) \| \| --- \| --- \|   **Study design**   \| X \| Individually-randomized parallel-group trial \| \| --- \| --- \| \| ⬜ \| Cluster-randomized parallel-group trial \| \| ⬜ \| Individually randomized cross-over (or other matched) trial \|   **For the purposes of this assessment, the interventions being compared are defined as**   \| Experimental: \| Dexamethasone \| Comparator: \| Saline placebo \| \| --- \| --- \| --- \| --- \|  \| **Specify which outcome is being assessed for risk of bias** \| Mortality \| \| --- \| --- \|  \| **Specify the numerical result being assessed.** In case of multiple alternative analyses being presented, specify the numeric result (e.g. RR = 1.52 (95% CI 0.83 to 2.77) and/or a reference (e.g. to a table, figure or paragraph) that uniquely defines the result being assessed. \| 60% mortality in experimental group vs 80% mortality in control group \| \| --- \| --- \|   **Is the review team’s aim for this result…?**   \| X \| to assess the effect of *assignment to intervention* (the ‘intention-to-treat’ effect) \| \| --- \| --- \| \| ⬜ \| to assess the effect of *adhering to intervention* (the ‘per-protocol’ effect) \|   **If the aim is to assess the effect of *adhering to intervention***, select the deviations from intended intervention that should be addressed (at least one must be checked):  ⬜ occurrence of non-protocol interventions  ⬜ failures in implementing the intervention that could have affected the outcome  ⬜ non-adherence to their assigned intervention by trial participants  **Which of the following sources were obtained to help inform the risk-of-bias assessment? (tick as many as apply)**  X Journal article(s) with results of the trial  ⬜ Trial protocol  ⬜ Statistical analysis plan (SAP)  ⬜ Non-commercial trial registry record (e.g. ClinicalTrials.gov record)  ⬜ Company-owned trial registry record (e.g. GSK Clinical Study Register record)  ⬜ “Grey literature” (e.g. unpublished thesis)  ⬜ Conference abstract(s) about the trial  ⬜ Regulatory document (e.g. Clinical Study Report, Drug Approval Package)  ⬜ Research ethics application  ⬜ Grant database summary (e.g. NIH RePORTER or Research Councils UK Gateway to Research)  ⬜ Personal communication with trialist  ⬜ Personal communication with the sponsor |
| --- | --- | --- | --- | --- | --- | --- | --- | --- | --- | --- | --- | --- | --- | --- | --- | --- | --- | --- | --- | --- |

## Risk of bias assessment

Responses underlined in green are potential markers for low risk of bias, and responses in red are potential markers for a risk of bias. Where questions relate only to sign posts to other questions, no formatting is used.

**Domain 1: Risk of bias arising from the randomization process**

| **Signalling questions** | **Comments** | **Response options** |
| --- | --- | --- |
| **1.1 Was the allocation sequence random?** | Not enough information. Study states double blind randomised trial with no further information | NI |
| **1.2 Was the allocation sequence concealed until participants were enrolled and assigned to interventions?** |  | NI |
| **1.3 Did baseline differences between intervention groups suggest a problem with the randomization process?** | Not stated | NI |
| **Risk-of-bias judgement** | Some concerns | Some concerns |
| Optional: What is the predicted direction of bias arising from the randomization process? | Unpredictable | Unpredictable |

Domain 2: Risk of bias due to deviations from the intended interventions (*effect of assignment to intervention*)

| **Signalling questions** | **Comments** | **Response options** |
| --- | --- | --- |
| **2.1. Were participants aware of their assigned intervention during the trial?** | Only stated that ‘double blind randomised’ however haemodynamically shocked patients would not be able to have process this information through a resuscitation | PN |
| **2.2. Were carers and people delivering the interventions aware of participants' assigned intervention during the trial?** |  | PN |
| **2.3. If Y/PY/NI to 2.1 or 2.2: Were there deviations from the intended intervention that arose because of the trial context?** |  |  |
| **2.4 If Y/PY to 2.3: Were these deviations likely to have affected the outcome?** |  |  |
| **2.5. If Y/PY/NI to 2.4: Were these deviations from intended intervention balanced between groups?** |  |  |
| **2.6 Was an appropriate analysis used to estimate the effect of assignment to intervention?** | Probably yes however not explicitly stated | PY |
| **2.7 If N/PN/NI to 2.6: Was there potential for a substantial impact (on the result) of the failure to analyse participants in the group to which they were randomized?** |  |  |
| **Risk-of-bias judgement** | Low risk | Low |
| Optional: What is the predicted direction of bias due to deviations from intended interventions? | NA | NA |

Domain 2: Risk of bias due to deviations from the intended interventions (*effect of adhering to intervention*)

| **Signalling questions** | **Comments** | **Response options** |
| --- | --- | --- |
| **2.1. Were participants aware of their assigned intervention during the trial?** |  | Y / PY / PN / N / NI |
| **2.2. Were carers and people delivering the interventions aware of participants' assigned intervention during the trial?** |  | Y / PY / PN / N / NI |
| **2.3. [If applicable:] If Y/PY/NI to 2.1 or 2.2: Were important non-protocol interventions balanced across intervention groups?** |  | NA / Y / PY / PN / N / NI |
| **2.4. [If applicable:] Were there failures in implementing the intervention that could have affected the outcome?** |  | NA / Y / PY / PN / N / NI |
| **2.5. [If applicable:] Was there non-adherence to the assigned intervention regimen that could have affected participants’ outcomes?** |  | NA / Y / PY / PN / N / NI |
| **2.6. If N/PN/NI to 2.3, or Y/PY/NI to 2.4 or 2.5: Was an appropriate analysis used to estimate the effect of adhering to the intervention?** |  | NA / Y / PY / PN / N / NI |
| **Risk-of-bias judgement** |  | Low / High / Some concerns |
| Optional: What is the predicted direction of bias due to deviations from intended interventions? |  | NA / Favours experimental / Favours comparator / Towards null /Away from null / Unpredictable |

Domain 3: Missing outcome data

| **Signalling questions** | **Comments** | **Response options** |
| --- | --- | --- |
| **3.1 Were data for this outcome available for all, or nearly all, participants randomized?** | Probably yes. No mention of missing data in study. | PY |
| **3.2 If N/PN/NI to 3.1: Is there evidence that the result was not biased by missing outcome data?** | Probably yes | PY |
| **3.3 If N/PN to 3.2: Could missingness in the outcome depend on its true value?** |  |  |
| **3.4 If Y/PY/NI to 3.3: Is it likely that missingness in the outcome depended on its true value?** |  |  |
| **Risk-of-bias judgement** | Low | Low |
| Optional: What is the predicted direction of bias due to missing outcome data? | NA | NA |

Domain 4: Risk of bias in measurement of the outcome

| **Signalling questions** | **Comments** | **Response options** |
| --- | --- | --- |
| **4.1 Was the method of measuring the outcome inappropriate?** | No | N |
| **4.2 Could measurement or ascertainment of the outcome have differed between intervention groups?** | No | N |
| **4.3 If N/PN/NI to 4.1 and 4.2: Were outcome assessors aware of the intervention received by study participants?** | Probably No | PN |
| **4.4 If Y/PY/NI to 4.3: Could assessment of the outcome have been influenced by knowledge of intervention received?** |  |  |
| **4.5 If Y/PY/NI to 4.4: Is it likely that assessment of the outcome was influenced by knowledge of intervention received?** |  |  |
| **Risk-of-bias judgement** | Low | Low |
| Optional: What is the predicted direction of bias in measurement of the outcome? | NA | NA |

Domain 5: Risk of bias in selection of the reported result

| **Signalling questions** | **Comments** | **Response options** |
| --- | --- | --- |
| **5.1 Were the data that produced this result analysed in accordance with a pre-specified analysis plan that was finalized before unblinded outcome data were available for analysis?** | NI | NI |
| **Is the numerical result being assessed likely to have been selected, on the basis of the results, from...** |  |  |
| **5.2. ... multiple eligible outcome measurements (e.g. scales, definitions, time points) within the outcome domain?** | No | NI |
| **5.3 ... multiple eligible analyses of the data?** | No | N |
| **Risk-of-bias judgement** | Some concerns | Some concerns |
| Optional: What is the predicted direction of bias due to selection of the reported result? | NA | NA |

Overall risk of bias

| **Risk-of-bias judgement** | Some concerns | Some concerns |
| --- | --- | --- |
| Optional: What is the overall predicted direction of bias for this outcome? | Unpredictable | Unpredictable |


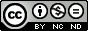


This work is licensed under a [Creative Commons Attribution-NonCommercial-NoDerivatives 4.0 International License](http://creativecommons.org/licenses/by-nc-nd/4.0/).
